# Tight genetic linkage of genes causing hybrid necrosis and pollinator isolation between young species

**DOI:** 10.1038/s41477-023-01354-8

**Published:** 2023-02-20

**Authors:** Chaobin Li, Marta Binaghi, Vivien Pichon, Gina Cannarozzi, Loreta Brandão de Freitas, Mathieu Hanemian, Cris Kuhlemeier

**Affiliations:** 1 Institute of Plant Sciences, University of Bern, Bern, Switzerland; 2 Department of Genetics, Laboratory of Molecular Evolution, Universidade Federal do Rio Grande do Sul, Porto Alegre, Brazil; 3 Laboratoire des Interactions Plantes-Microbes-Environnement (LIPME), INRAE, CNRS, Université de Toulouse, Castanet-Tolosan, France; 4 Present Address: Department of Biology, University of Fribourg, Fribourg, Switzerland; 5 Present Address: Chemistry/Biology/Pharmacy Information Center, ETH Zürich, Zürich, Switzerland

**Keywords:** Plant immunity, Plant evolution

## Abstract

The mechanisms of reproductive isolation that cause phenotypic diversification and eventually speciation are a major topic of evolutionary research. Hybrid necrosis is a post-zygotic isolation mechanism in which cell death develops in the absence of pathogens. It is often due to the incompatibility between proteins from two parents. Here we describe a unique case of hybrid necrosis due to an incompatibility between loci on chromosomes 2 and 7 between two pollinator-isolated *Petunia* species. Typical immune responses as well as endoplasmic reticulum stress responses are induced in the necrotic line. The locus on chromosome 2 encodes ChiA1, a bifunctional GH18 chitinase/lysozyme. The enzymatic activity of ChiA1 is dispensable for the development of necrosis. We propose that the extremely high expression of ChiA1 involves a positive feedback loop between the loci on chromosomes 2 and 7. *ChiA1* is tightly linked to major genes involved in the adaptation to different pollinators, a form of pre-zygotic isolation. This linkage of pre- and post-zygotic barriers strengthens reproductive isolation and probably contributes to rapid diversification and speciation.

## Abstract (web summary)

A chitinase gene is responsible for the hybrid necrosis between two young *Petunia* species. This gene is tightly linked with genes in pollinator-mediated reproductive isolation, suggesting cooperation of pre- and post-zygotic barriers.

## Main

Speciation depends on the evolution of reproductive barriers that reduce gene flow between previously interbreeding populations^1–3^. In plants, pollinator-mediated isolation constitutes a crucial pre-zygotic isolation barrier^4^. The preferences of distinct classes of pollinators for sets of floral traits such as colour, morphology, scent and nectar production are considered a driving force in the rapid diversification of the angiosperms^5^. These matching sets of floral traits are called pollination syndromes. However, pollinator preference for a flower type is rarely absolute, and hybridization between (incipient) species is often observed. Thus, plant speciation generally involves the accumulation of multiple reproductive barriers^3^.

In plants, hybrid necrosis (HN) is a post-zygotic isolation barrier in which hybrids show necrotic leaves and poor growth^6,7^. Many of the reported cases were caused by deleterious epistatic interactions between alleles at two or more loci, creating a post-mating barrier that reduces fitness in the hybrids but not in the parents^8^. HN often involves genes encoding components of the immune system^9,10^. For instance, many reported cases of HN in *Arabidopsis* are due to improper combinations of nucleotide-binding domain leucine-rich repeat (NLR) proteins, which are key players in the immune systems of both plants and vertebrates^11–14^.

We have previously studied the genetic basis of the evolution of pollination syndromes in the South American genus *Petunia* (Solanaceae). *Petunia axillaris* (*P. axillaris*) is widely distributed in southern South America, while *Petunia exserta* (P. exserta) is a highly endemic species that is found exclusively in shaded shelters in the Guaritas Region of Rio Grande do Sul, Brazil^15^. *P. axillaris* has white, ultraviolet (UV)-absorbent, fragrant flowers and is pollinated by hawkmoths^16,17^, while *P. exserta* has red, scentless flowers with exserted stamens and stigma and is pollinated by hummingbirds^15,18^. Single genes underlying major quantitative trait loci (QTLs) for UV absorption (*MYB-FL*), scent production (*CNL1*) and pistil length (*EOBII*) were found to be tightly linked in a so-called supergene region on chromosome 2^19–22^. Such tight genetic linkage is thought to impede the dissolution of pollination syndromes by recombination. The two species are thought to have evolved recently from a common ancestor that was most likely hawkmoth pollinated^23–25^. Natural hybrids are present in a few locations in the Guaritas Region where the two species are sympatric, showing that reproductive barriers are incomplete^18,25,26^.

In this Article, we describe a case of HN in a cross between *P. axillaris* and *P. exserta*. The necrotic symptoms are associated with a deleterious combination between two loci located on chromosome 2 and chromosome 7. We show that the locus on chromosome 2 encodes ChiA1, a bifunctional chitinase/lysozyme with a major role in pattern-triggered immunity against fungi and bacteria^27^. We discuss the potential relevance of the tight linkage of *ChiA1* to pre-zygotic isolation barriers.

## Results

### An introgression line shows necrotic symptoms

**Fig. 1:**
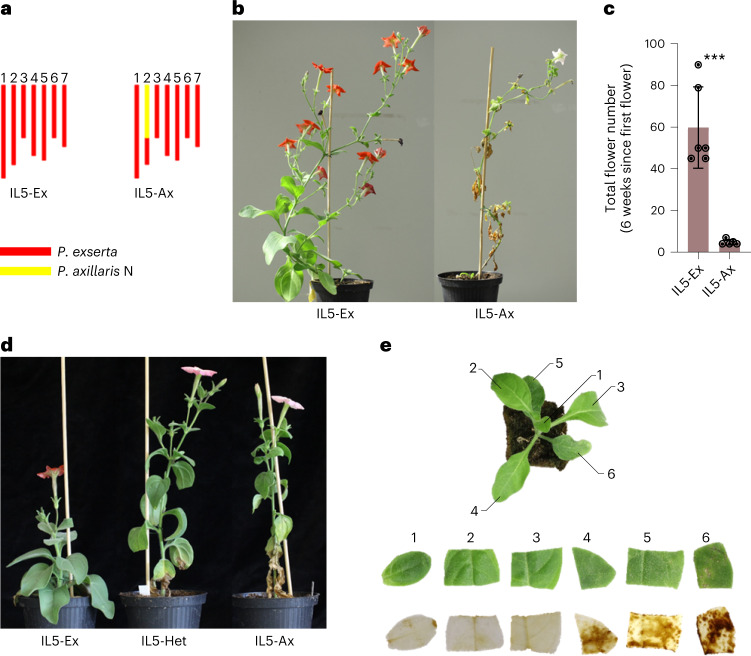
IL5 shows necrotic symptoms. **a**, Diagram showing the genotypes of the IL5 homozygous versions. Yellow colour indicates the *P. axillaris* N homozygous region, and red colour indicates the *P. exserta* homozygous region. The seven chromosomes are shown in a left-to-right order. **b**, Pictures of representative IL5-Ex and IL5-Ax plants 13 weeks after sowing. **c**, Flower production analysis in IL5-Ex and IL5-Ax plants. Total flower numbers were recorded during 6 weeks after the development of the first flower (~10 weeks after sowing) in each genotype. Data are presented as mean ± standard deviation (s.d.) (*n* = 5 for IL5-Ax plants; *n* = 6 for IL5-Ex plants). ***P < 0.001; two-sided Student’s *t*-test (*P* = 0.0002). **d**, Pictures of representative IL5-Ex, IL5-Het and IL5-Ax plants 10 weeks after sowing. **e**, Assessm**e**nt of ROS production using DAB staining of a representative 5-week-old IL5-Ax plant. DAB was oxidized by hydrogen peroxide (ROS) and stained in brown. The experiment was repeated twice with similar results (*n* = 3). Source data

**Extended Data Fig. 1:**
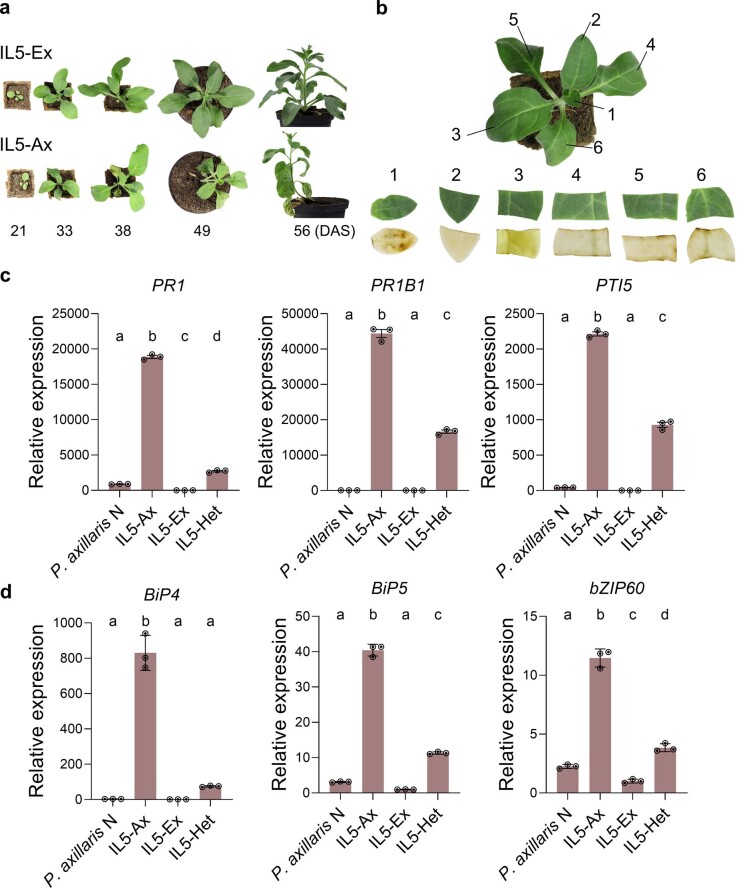
Characterization of IL5-Ax plants. **a**, Plant phenotype of IL5-Ax and IL5-Ex at different days after sowing (DAS). **b**, Assessment of ROS production using DAB staining of a representative 5 weeks old IL5-Ex plant. DAB was oxidized by hydrogen peroxide (ROS) and stained in brown. The experiment was repeated once with similar results (*n* = 2). **c**, Quantitative RT-PCR showing the expression levels of pathogenesis-related genes *PR1*, *PR1B1*, and *PTI5* in leaves of 10 weeks old *P. axillaris* N, IL5-Ax, IL5-Ex, and IL5-Het plants. Data are presented as mean values ± s.d. of three biological replicates (*n* = 3). Different letters indicate significant differences (*P* < 0.05, one-way ANOVA, Tukey’s HSD test); For *P* values, see Source Data. **d**, Quantitative RT-PCR showing the expression levels of ER-stress marker genes *BiP4*, *BiP5*, and *bZIP60* in leaves of 10 weeks old *P. axillaris* N, IL5-Ax, IL5-Ex, and IL5-Het plants. Data are presented as mean values ± s.d. of three biological replicates (*n* = 3). Different letters indicate significant differences (*P* < 0.05, one-way ANOVA, Tukey’s HSD test); for *P* values, see Source Data. Source data

The introgression line IL5 is derived from a cross between hawkmoth-pollinated *P. axillaris* N and hummingbird-pollinated *P. exserta*^28^. It segregates for a *P. axillaris* region of low recombination on chromosome 2 in an otherwise *P. exserta* background (Fig. 1a). Several major genes affecting pollination syndrome traits were previously identified in this so-called supergene region^19–22^. Unexpectedly, the progenies of IL5 that are homozygous for *P. axillaris* N at the supergene region (hereafter called IL5-Ax) displayed necrotic symptoms that strongly affect plant growth (Fig. 1b) and flower production (Fig. 1c). IL5-Ex (homozygous for *P. exserta* at the supergene region) plants were healthy. The necrotic symptoms in IL5-Ax can be observed as early as 38 days after sowing (Extended Data Fig. 1a). The symptoms were also observed in plants heterozygous for the introgression (IL5-Het) but were milder than in the IL5-Ax plants, suggesting semi-dominance (Fig. 1d). Necrosis was associated with the production of reactive oxygen species (ROS) and increased levels of expression of key pathogenesis-related genes (Fig. 1e and Extended Data Fig. 1b,c). These data are consistent with HN, in which an immune response is triggered in the absence of pathogens^10^. Endoplasmic reticulum (ER) stress marker genes^29^, for example, two luminal binding protein coding genes *BiP4* and *BiP5*, and transcription factor *bZIP60*, were highly induced in the necrotic line (Extended Data Fig. 1d), suggesting ER-stress-induced cell death may play a role in HN development^30^.

### HN is caused by genetic interaction of two loci

**Fig. 2:**
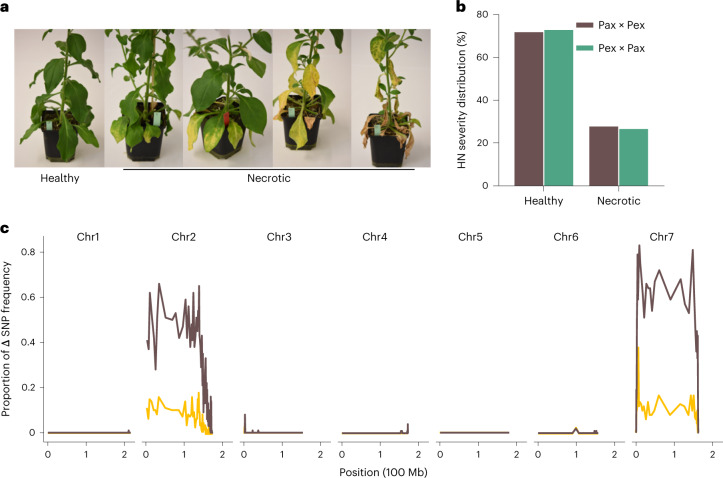
Genetic mapping of HN between *P. axillaris* N and *P. exserta*. **a**, Typical phenotype of the F2 plants 13 weeks after sowing. The plants were classified into healthy or necrotic groups. **b**, Distribution of the F2 plants qualitatively classified according to **a**. A total of 384 plants from each of the F2 populations were phenotyped. Pax*, P. axillaris* N; Pex, *P. exserta*. **c**, QTL mapping using bulked segregant RNA-seq method showing regions with excessive difference in allele frequency between the pool of necrotic and the pool of healthy individuals. A high value on the *Y* axis indicates that many of the SNP positions in a window present an allele frequency difference outside of the genome-wide quantile thresholds. Thresholds are set at genome-wide quantiles 0.05 and 0.95 (brown line) and 0.01 and 0.99 (yellow line). Stepping windows include 100 SNPs.

**Extended Data Fig. 2:**
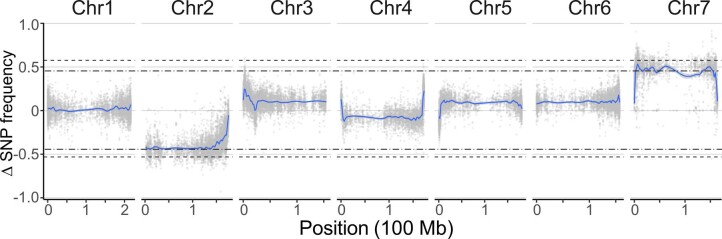
Raw ΔSNP frequency between the necrotic and healthy group for BSR-seq. Dots represent the difference in frequency between the groups for each SNP. A y value higher than 0 indicates a higher frequency of the *P. exserta* allele in the necrotic group. This trend is observed in large part of Chr 7. A y value lower than 0 indicates a higher frequency of the *P. axillaris* N allele in the necrotic group. This trend is observed in large part of Chr 2. Blue lines represent the Loess smoothing, with 0.95 confidence intervals represented in light grey. Dashed lines indicate 0.01 and 0.99 genome-wide quantile value, while dash-dotted lines indicate the 0.05 and 0.95 quantile value.

HN often results from incompatibilities between genetic loci that have diverged over time and are brought together in hybrids^10^. Such interactions can involve pairs of nuclear genes or nucleo-cytoplasmic interactions^31,32^. Therefore, we generated reciprocal F2 populations derived from *P. axillaris* N and *P. exserta* to dissect the genetic architecture of HN and identify the loci interacting with the chromosome 2 region. Both populations displayed comparable distributions of HN scores, that is, approximately three-quarters of the plants did not show symptoms and one-quarter of the plants displayed symptoms. The similar distribution of HN scores in the two populations indicates that the loci controlling HN are nuclear, as cytoplasmic inheritance is unidirectional (Fig. 2a,b). Bulked segregant RNA sequencing (BSR-seq) was used to locate the genomic regions causing HN. Strong signals associated with the necrotic phenotype were detected on chromosomes 2 and 7 (Fig. 2c and Extended Data Fig. 2). We named these loci *HNe2* and *HNe7*. The necrosis is triggered by a combination of *P. axillaris* N alleles at *HNe2* and *P. exserta* alleles at *HNe7* (Fig. 2c and Extended Data Fig. 2) in accordance with the genetic make-up of IL5-Ax (Fig. 1a).

### Map-based cloning of *HNe2*

**Fig. 3:**
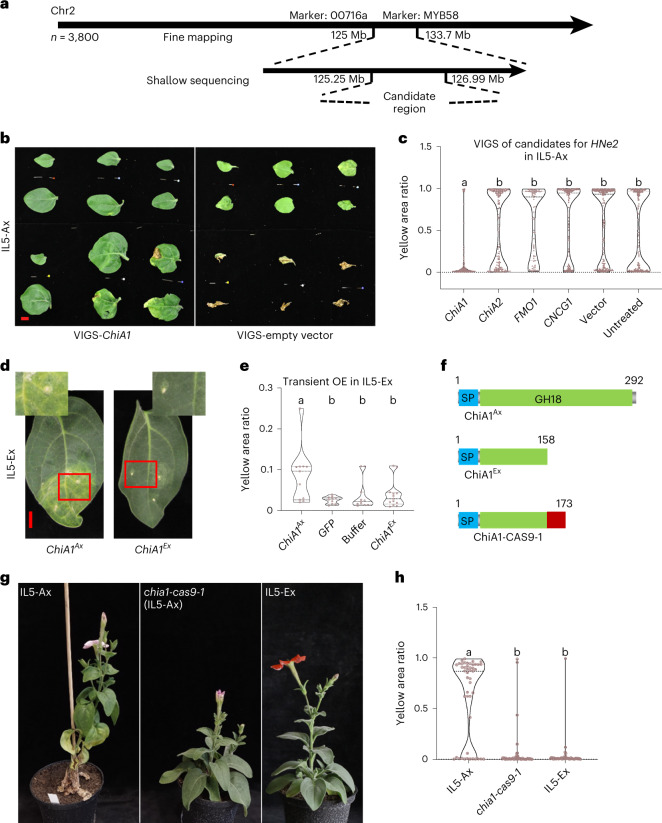
*HNe2* encodes a chitinase ChiA1. **a**, Diagram showing the map-based cloning of *HNe2*. Firstly, the *HNe2* locus was mapped to an 8.7 Mb region on chromosome 2. For details, see Methods and Supplementary Tables 1–4. **b**,**c**, VIGS of the four selected candidate genes for *HNe2*: representative leaf phenotypes from one branch arising after the *ChiA1* VIGS treatment and empty control VIGS treatment in IL5-Ax; leaves are shown from a top-to-bottom order; scale bar, 1 cm (**b**). Yellow area ratio of IL5-Ax plant leaves after VIGS of *ChiA1*, *ChiA2*, *FMO1*, *CNCG1* and empty control; five biological replicates were used for each treatment (*n* = 5 plants). Symptoms were quantified on the basis of the ratio of the area with yellow colour compared with the whole leaf area. A higher value indicates a higher proportion of yellow surface. Different letters indicate significant differences (*P* < 0.05, one-way ANOVA, Tukey’s honestly significant difference (HSD) test); for *P* values, see source data (**c**). **d**,**e**, Transient overexpression of *ChiA1*^*Ax*^ in IL5-Ex plant leaves: A representative phenotype of IL5-Ex leaf after agro-infiltration with 35S::*ChiA1*^*Ax*^ or 35S::*ChiA1*^*Ex*^ as a control; scale bar, 1 cm (**d**). Yellow area ratio of IL5-Ex plant leaves after agro-infiltration of 35S::*ChiA1*^*Ax*^, 35S::*ChiA1*^*Ex*^, 35S::*GFP* and empty infiltration buffer. Three individual plants were used for each treatment. *ChiA1*^*Ex*^, *GFP* and infiltration buffer were used as negative controls. The phenotype was analysed 2 weeks after the infiltration. Different letters indicate significant differences (*P* < 0.05, one-way ANOVA, Tukey’s HSD test); for *P* values, see source data (**e**). **f**, Diagram showing the predicted protein products of *ChiA1* gene in IL5-Ax, IL5-Ex and *chia1-cas9-1*. Red box indicates the mismatched protein sequence caused by frameshift. The numbers indicate the amino acid sites. SP, signal peptide; GH18, glycosyl hydrolase 18 domain. **g**,**h**, Plant phenotype (**g**) and leaf yellow area ratio (**h**) of *chia1-cas9-1* in IL5-Ax (middle) compared with IL5-Ax (left) and IL5-Ex (right). For the leaf yellow area ratio, three individual plants were analysed for each genotype. Different letters indicate significant differences (*P* < 0.05, one-way ANOVA, Tukey’s HSD test); for *P* values, see source data. Source data

**Extended Data Fig. 3:**
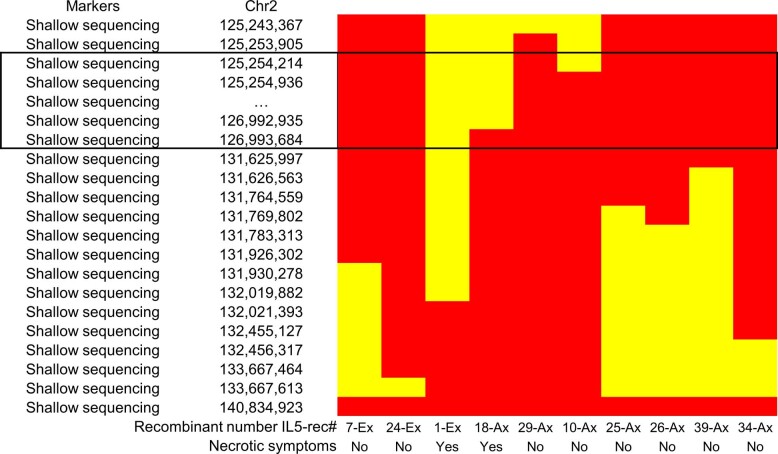
Positional cloning using shallow sequencing of ten informative recombinant lines. Schematic showing the genotypes of the most informative SNP markers obtained from shallow sequencing of ten informative recombinant lines (Fig. 3a and Supplementary Table 1). Red color indicates the genotype of the sample at the specified SNP is homozygous for *P. exserta*; yellow color indicates the genotype of a sample at the specified SNP is homozygous for *P. axillaris* N. The black frame indicates the region associated with the HN phenotype in the fine mapping.

To reduce the interval carrying *HNe2*, a fine-mapping approach was undertaken through the search of recombinant progenies of IL5-Het. The relationship between the phenotypic segregation of HN and the genotype of the recombinant lines led to a first interval of 8.7 Mb (Fig. 3a and Supplementary Table 1). Then, the recombination breakpoints of ten of the most informative lines were precisely characterized through whole-genome sequencing, resulting in a 1.74 Mb interval (Fig. 3a and Extended Data Fig. 3). As this region still contains 60 genes (Supplementary Table 2), an RNA sequencing (RNA-seq) analysis of leaf tissues from IL5-Ax and IL5-Ex plants was performed to investigate their functional relevance on the basis of expression. Among the 34 expressed genes in the candidate region (read counts >100 in at least one of the samples; Supplementary Table 3), 8 were differentially expressed (*q* < 0.001) including 4 genes known to play a role in plant–pathogen interactions: *ChiA1*, *ChiA2*, *FMO1* and *CNGC1* (Table 1 and Supplementary Table 4).

**Table 1:** List of the four best candidate genes underlying *HNe2*. DEGs that are immune response related were considered. Gene ID, annotation, expression (normalized read counts), *q* value and alignment results are shown. The en dash indicates no protein sequence change or only amino acid changes found between *P. axillaris* N and *P. exserta*. Gene ID and scientific names of the species are italicized

Gene ID	Annotation	IL5-Ax	IL5-Ex	*q* value	Remark
*Peaxi162Scf00403g00840.1*	ChiA1 Acidic endochitinase	42,950.59	21.93	2.92 × 10^−52^	Nonsense mutation in *P. exserta*
*Peaxi162Scf00191g01019.1*	FMO1 Probable flavin-containing monooxygenase 1	1,171.69	0.98	2.03 × 10^−42^	Duplicated in *P. exserta*
*Peaxi162Scf00403g00838.1*	ChiA2 Acidic endochitinase	155.31	0.00	2.1 × 10^−14^	–
*Peaxi162Scf00403g00524.1*	CNGC1 Cyclic nucleotide-gated ion channel 1	4,284.78	613.87	3.32 × 10^−6^	–

**Extended Data Fig. 4:**
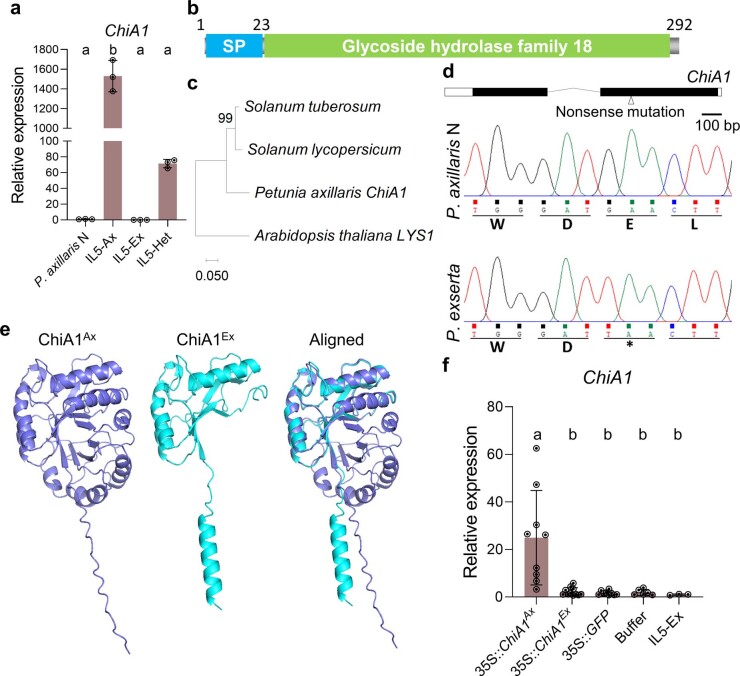
Expression analysis, domain annotation, and phylogenetic analysis of *ChiA1* and confirmation of the nonsense mutation of *ChiA1* in *P. exserta*. **a**, Quantitative RT-PCR showing the transcription of *ChiA1* in *P. axillaris* N, IL5-Ax. IL5-Ex, and IL5-Het leaves. Data are presented as mean values ± s.d. of three biological replicates (*n* = 3). Different letters indicate significant differences (*P* < 0.05, one-way ANOVA, Tukey’s HSD test); for *P* values, see Source Data. **b**, Domain annotation of ChiA1 suggesting it is a glycoside hydrolase (GH18) bifunctional chitinase with a secretion signal peptide (SP). **c**, Phylogenetic relationships of *ChiA1* and its homologs in some other species. Distances were estimated using the neighbor-joining algorithm. The numbers at the nodes represent their confidence level, shown as a percentage from 1,000 bootstraps. The scale bar indicates the average number of amino acid substitutions per site. The *Arabidopsis thaliana* homolog was used as an outgroup. **d**, Nonsense mutation in *ChiA1 P. exserta* allele. The upper panel shows the schematic representation of *ChiA1* with the G to T nonsense mutation. The mutated site is indicated by a triangle. Thin line indicates the intron. Black boxes indicate the exons. White boxes indicate the untranslated regions. The lower panel shows the Sanger sequencing confirming the G to T mutation of *ChiA1* in *P. exserta*. Asterisk indicates the translation stop codon. **e**, Structure models of *ChiA1*^*Ax*^ and *ChiA1*^*Ex*^ predicted by AlphaFold. Protein sequences of *ChiA1*^*Ax*^ and *ChiA1*^*Ex*^ are used for the structure prediction. **f**, Quantification of *ChiA1* expression levels in IL5-Ex plant leaves transiently overexpressing *ChiA1*^*Ax*^, *ChiA1*^*Ex*^, *GFP*, or negative controls. Data are presented as mean values ± s.d. (*n* = 9 leaves for each treatment). Different letters indicate significant differences (*P* < 0.05, one-way ANOVA, Tukey’s HSD test); for *P* values, see Source Data. Source data

*Chitinase A1* (*ChiA1*) is one of the most highly expressed genes in IL5-Ax (Table 1 and Extended Data Fig. 4a) and is annotated as a bifunctional endochitinase^33^ (Extended Data Fig. 4b). The protein encoded by *ChiA1* is homologous to AtLYS1/ChiA (AT5G24090), which has a central role in triggering immune responses in *Arabidopsis*^27^ (Extended Data Fig. 4c). Importantly, *ChiA1* carries a nonsense mutation in *P. exserta*, which leads to a truncated protein of 158 amino acids (Extended Data Fig. 4d,e). *ChiA2* is closely related to *ChiA1* but shows a much lower expression level than *ChiA1* in IL5-Ax (Table 1). *Flavin-containing monooxygenase 1* (*FMO1*) plays key roles in systemic acquired resistance by synthesizing N-OH-Pip from pipecolic acid^34^. It has two copies in the *P. exserta* genome, whereas only one copy was present in *P. axillaris* N. *Cyclic nucleotide-gated ion channel 1* (*CNGC1*) belongs to a gene family involved in plant immunity by triggering calcium signalling and hypersensitive response^35,36^.

**Extended Data Fig. 5:**
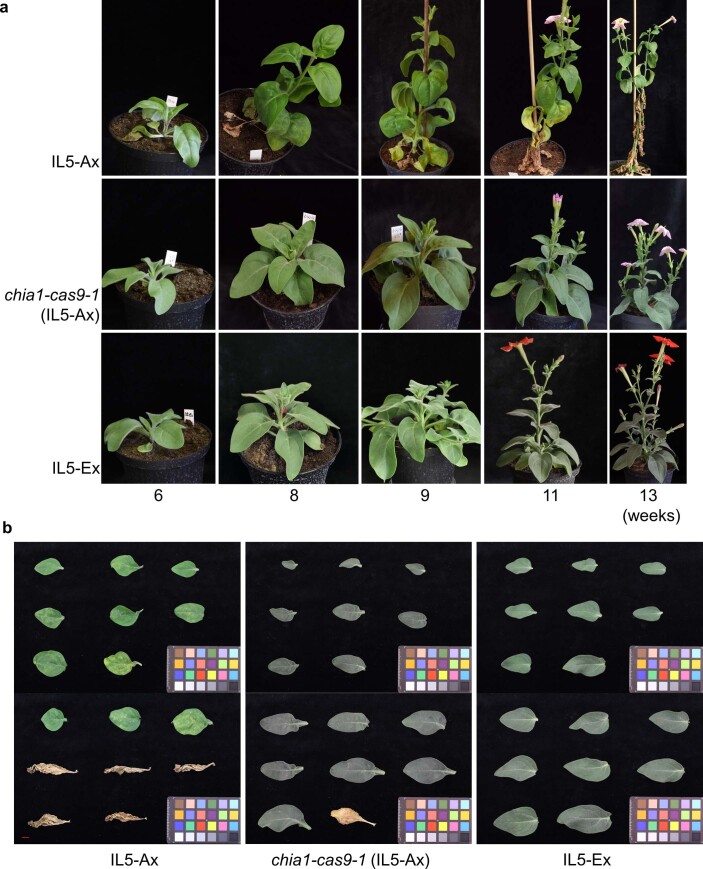
Detailed phenotype of *chia1-cas9-1* in IL5-Ax compared with IL5-Ax and IL5-Ex. **a**, Plant phenotype of *chia1-cas9-1* in IL5-Ax compared with IL5-Ax and IL5-Ex at different weeks after sowing. **b**, Leaf phenotype of *chia1-cas9-1* in IL5-Ax compared with IL5-Ax and IL5-Ex. The leaf phenotype was photographed at 13 weeks after sowing. Bar, 1 cm.

To functionally validate these four candidates for *HNe2*, we knocked down their transcript levels in IL5-Ax by virus-induced gene silencing (VIGS)^37^. VIGS of *ChiA1* caused a robust decrease of necrosis in IL5-Ax plants compared with the empty vector or untreated controls (Fig. 3b,c). In contrast, VIGS of *ChiA2*, *FMO1* and *CNGC1* showed no significant reductions of symptoms (Fig. 3c). To confirm the role of ChiA1 in HN, we transiently overexpressed *ChiA1*^*Ax*^ (*P. axillaris* N allele) as well as *ChiA1*^*Ex*^ (*P. exserta* allele) in IL5-Ex leaves driven by the 35S promoter. *ChiA1*^*Ax*^ was highly expressed, while *ChiA1*^*Ex*^ was barely detectable (Extended Data Fig. 4f). Only leaves overexpressing *ChiA1*^*Ax*^ turned yellow in the region of infiltration (Fig. 3d,e). Thus, the lack of messenger RNA accumulation is post-transcriptional, most likely due to nonsense-mediated decay (NMD)^38^. Furthermore, we generated a *ChiA1* loss-of-function mutant in the IL5-Ax background by clustered regularly interspaced short palindromic repeats (CRISPR)–Cas9. The mutant (*chia1-cas9-1*) has a 4 bp deletion, leading to a frameshift and a premature stop codon, resulting in a truncated protein of 173 amino acids (instead of the 292 amino acid full length), similar to the protein encoded by *ChiA1*^*Ex*^ (Fig. 3f). The necrotic symptoms were strongly reduced, and plant morphology was similar to IL5-Ex (Fig. 3g,h and Extended Data Fig. 5a,b). The combined results prove beyond reasonable doubt that *ChiA1* is the gene underlying *HNe2*.

### HN does not require ChiA1 enzymatic activity

**Fig. 4:**
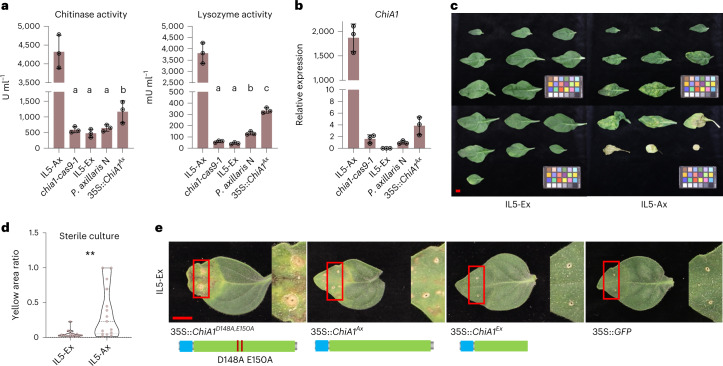
Necrotic symptoms do not depend on pathogens or ChiA1’s glycoside hydrolase activity. **a**, Chitinase and lysozyme activity in leaf tissues of IL5-Ax, *chia1-cas9-1* (IL5-Ax), IL5-Ex, *P. axillaris* N and *ChiA1* overexpression line in *P. axillaris* N background (35S::*ChiA1*^*Ax*^). For each genotype, nine plants were used and were grouped into three for replicates. Sixty-six leaf discs were sampled from each group to unify the total leaf area used. Data are presented as mean ± s.d. Different letters indicate significant differences (*P* < 0.05, one-way ANOVA, Tukey’s honestly significant difference (HSD) test); IL5-Ax was not included in the ANOVA test; for *P* values, see source data. **b**, Quantitative RT–PCR showing the transcription of *ChiA1* in leaves of 12-week-old IL5-Ax, *chia1-cas9-1* (IL5-Ax), IL5-Ex, *P. axillaris* N and *ChiA1* overexpression line in *P. axillaris* N (35S::*ChiA1*) background. Data are presented as mean ± s.d. of three biological replicates (*n* = 3). **c**, Representative leaf phenotypes of IL5-Ax and IL5-Ex plants in sterile culture. The phenotype of the leaves was analysed 12 weeks after sowing. Four individual plants of each genotype were observed with similar results. Scale bar, 1 cm. **d**, Necrosis severity analysis of IL5-Ax and IL5-Ex plant leaves from **c**. The yellow area ratio is based on the ratio of the area with yellow colour compared with the whole leaf area. A higher value indicates a larger yellow area ratio. *n* = 15 leaves for IL5-Ax and 16 leaves for IL5-Ex. (**P < 0.01, two-sided Student’s *t*-test, *P* = 0.0023). **e**, Representative phenotype of IL5-Ex leaves transiently overexpressing *ChiA1*^*D148A*, *E150A*^, *ChiA1*^*Ax*^, *ChiA1*^*Ex*^ or *GFP*. The 35S promoter was used for the overexpression. Seven-week-old IL5-Ex plant leaves were used for agro-infiltration. Photos were taken 2 weeks after the infiltration. Three biological replicates were used with similar results (Extended Data Fig. 7b). Scale bar, 1 cm. Schematics of the protein products of the ChiA1 variants are shown in the lower panel. Source data

ChiA1 harbours the glycoside hydrolase 18 (GH18) domain and a secretion signal peptide (Extended Data Fig. 4b) and is probably a bifunctional enzyme digesting both chitin and peptidoglycan as the homologous proteins in rubber tree (*Hevea brasiliensis*) and *Arabidopsis*^27,39^. We observed that chitinase and lysozyme activities were extremely high in IL5-Ax leaves compared with the other lines (Fig. 4a). In the *chia1-cas9-1* mutant, these activities were reduced to a level similar to that in IL5-Ex. *P. axillaris* N plants overexpressing *ChiA1* (35S::*ChiA1*) showed higher activity levels compared with wild-type *P. axillaris* N. Moreover, the enzyme activities were in line with the transcription levels of *ChiA1* (Fig. 4b). These results indicate that ChiA1 is responsible for the high chitinase and lysozyme activities in IL5-Ax leaves.

**Extended Data Fig. 6:**
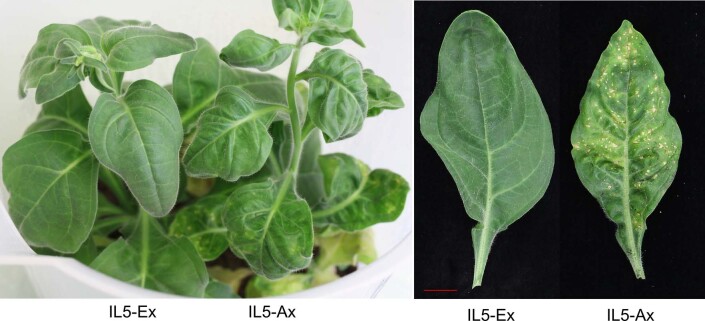
Detailed phenotype of IL5-Ax and IL5-Ex in sterile culture. Sterile culture of IL5-Ax and IL5-Ex in a sealed transparent beaker (left) and one representative leaf from each of IL5-Ex and IL5-Ax plants in sterile culture (right). Bar, 1 cm.

**Extended Data Fig. 7:**
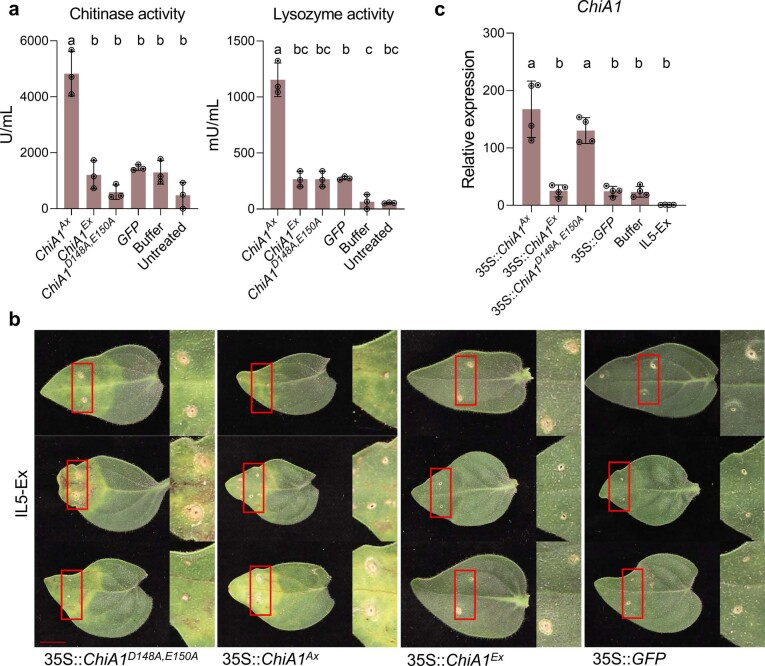
ChiA1 catalytic activity is not required for necrosis induction. **a**. Chitinase and lysozyme activity in leaf tissues transiently overexpressing *ChiA1*^*Ax*^, *ChiA1*^*Ex*^, or *ChiA1*^*D148A*, *E150A*^ in 7 weeks old IL5-Ex background, with the overexpression of *GFP*, infiltration buffer, or untreated plant leaves as controls. For each genotype, 9 plants were used and were grouped into 3 for replicates. Sixty-six leaf discs were sampled from each group to unify the total leaf area used. Data are presented as mean values ± s.d. Different letters indicate significant differences (*P* < 0.05, one-way ANOVA, Tukey’s HSD test); for *P* values, see Source Data. **b**, Phenotype of IL5-Ex leaves transiently overexpressing *ChiA1*^*D148A*, *E150A*^, *ChiA1*^*Ax*^, *ChiA1*^*Ex*^, and *GFP* with three biological replicates. The 35S promoter was used for the overexpression. *ChiA1*^*Ex*^ and *GFP* were used as controls. 7 or 8 weeks old IL5-Ex plant leaves were used for agro-infiltration. Photos were taken 2 weeks after the infiltration. Bar, 1 cm. **c**. Quantification of *ChiA1* expression levels in IL5-Ex plant leaves transiently overexpressing *ChiA1*^*Ax*^, *ChiA1*^*Ex*^, *ChiA1*^*D148A*, *E150A*^, *GFP*, or negative controls. Data are presented as mean values ± s.d. (*n* = 4 leaves for each treatment). Different letters indicate significant differences (*P* < 0.05, one-way ANOVA, Tukey’s HSD test); for *P* values, see Source Data. Source data

As ChiA1 degrades microbial cell wall components, we asked whether the necrotic symptoms might be caused by conserved degradation products of microbial cell walls that could act as microbe-associated molecular patterns^40^. When grown under axenic conditions, IL5-Ax plants still developed strong necrotic symptoms (Fig. 4c,d; and Extended Data Fig. 6). This suggests that necrotic symptoms develop in the absence of a microbial substrate for ChiA1. To test whether symptom development relied on ChiA1 enzymatic activity, we generated a *ChiA1* mutant in which the critical amino acids D148 and E150 in the glycoside hydrolase active site DXDXE motif are replaced by alanine (Fig. 4e). Mutation of these two amino acids abolishes both chitinase and lysozyme activities^41–43^ (Extended Data Fig. 7a). IL5-Ex leaves overexpressing either the catalytically inactive *ChiA1*^*D148A*, *E150A*^ or *ChiA1*^*Ax*^ showed strong necrosis at the site of infiltration, whereas the overexpression of *ChiA1*^*Ex*^ and *GFP* did not induce symptoms (Fig. 4e and Extended Data Fig. 7b,c). These results show that the necrotic symptoms represent a case of autoimmunity that does not rely on the enzymatic activities of ChiA1.

### ChiA1-dependent induction of WRKY18 induces necrotic symptoms

**Fig. 5:**
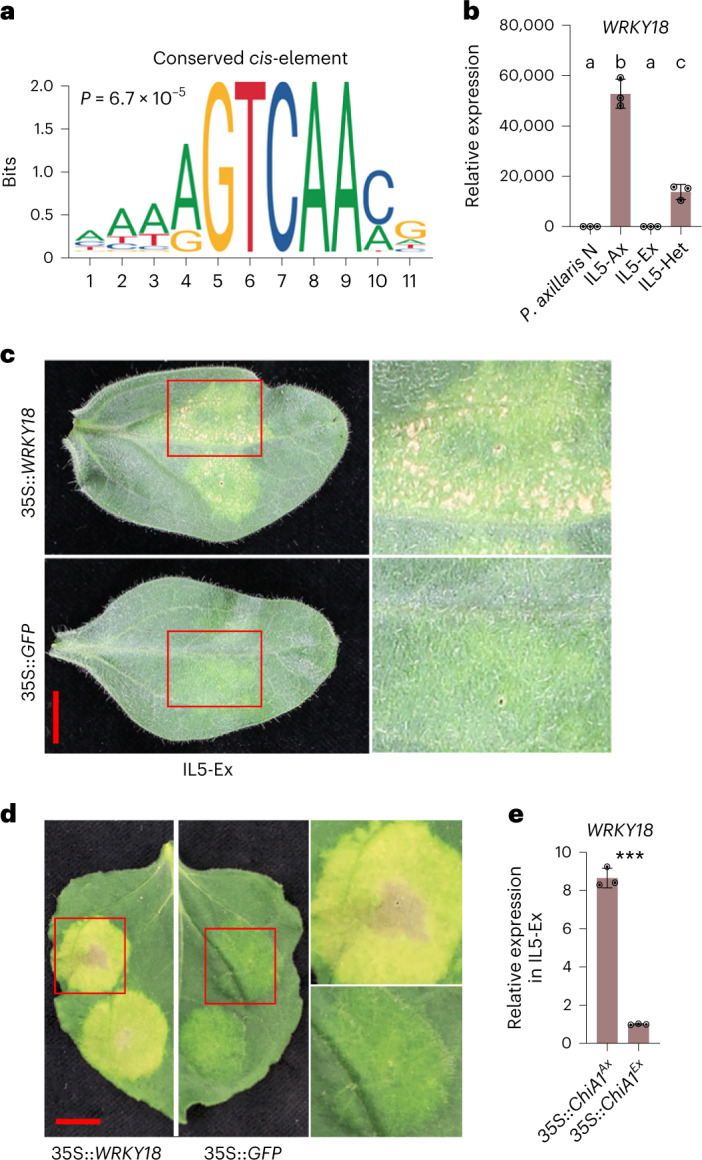
*WRKY18* overexpression induces necrotic symptoms and can be induced by ChiA1. **a**, The conserved *cis*-element found in the promoters of the DEGs between IL5-Ax and IL5-Ex leaf tissues. One-kilobase promoter sequences upstream of translation start codon of the 1717 DEGs were used for the *cis*-element analysis. **b**, Quantitative RT–PCR showing the transcription levels of *WRKY18* in *P. axillaris* N, IL5-Ax, IL5-Ex and IL5-Het plant leaves. Data are presented as mean ± s.d. of three biological replicates (*n* = 3). Different letters indicate significant differences (*P* < 0.05, one-way ANOVA, Tukey’s honestly significant difference (HSD) test); for *P* values, see source data. **c**, Representative pictures of IL5-Ex leaves 2 weeks after agro-infiltration carrying 35S::*WRKY18*, with 35S::*GFP* as a control. Five biological replicates were observed with similar phenotype. The right panels are the close-ups of the regions with red frames. Scale bar, 1 cm. **d**, A representative picture of the tobacco (*Nicotiana benthamiana*) leaves 5 days after agro-infiltration carrying 35S::*WRKY18*, with 35S::*GFP* as a control. Three biological replicates were observed with similar phenotype. Five-week-old tobacco leaves were used for agro-infiltration. The right panels are the close-ups of the regions with red frames. Scale bar, 1 cm. **e**, Quantitative RT–PCR showing the transcription levels of *WRKY18* in leaf tissues overexpressing *ChiA1*^*Ax*^ or *ChiA1*^*Ex*^ as shown in Fig. 3d. Data are presented as mean ± s.d. of three biological replicates (*n* = 3). ***P < 0.001; two-sided Student’s *t*-test (*P* = 0.0000). Source data

Transcriptional reprogramming is an integral part of plant immunity^44^. To better understand the mechanism underlying the activation of the immune system in IL5-Ax, we screened the promoters of the 1,717 differentially expressed genes (DEGs; *q* < 0.001) between IL5-Ax and IL5-Ex for conserved *cis*-elements (Supplementary Table 5). A WRKY binding site (Fig. 5a) was present in 60% of the DEG promoters (Supplementary Table 6). WRKY transcription factors are involved in pathogen responses, abiotic stress responses and senescence^45,46^. Of all the DEGs encoding WRKY transcription factors, *WRKY18* (homologous to At*WRKY18*) showed the highest expression level in IL5-Ax, while its expression was negligible in IL5-Ex or *P. axillaris* N wild-type plant leaves (Fig. 5b). Moreover, AtWRKY18 binds to the typical binding motif in its target genes^47^ (Fig. 5a). Overexpression of *WRKY18* induced necrotic symptoms in leaves of IL5-Ex as well as *Nicotiana benthamiana* (Fig. 5c,d). Importantly, agro-infiltration of 35S::*ChiA1*^*Ax*^ but not 35S::*ChiA1*^*Ex*^ in IL5-Ex leaves induced *WRKY18* expression (Figs. 5e and 3d and Extended Data Fig. 4f). We conclude that the massive transcriptional reprogramming observed in IL5-Ax is mediated through ChiA1-dependent induction, most likely indirectly, of one or more WRKY18-type transcription factors.

### The synteny of *ChiA1* and *MYB-FL* is specific to *Petunia*

**Fig. 6:**
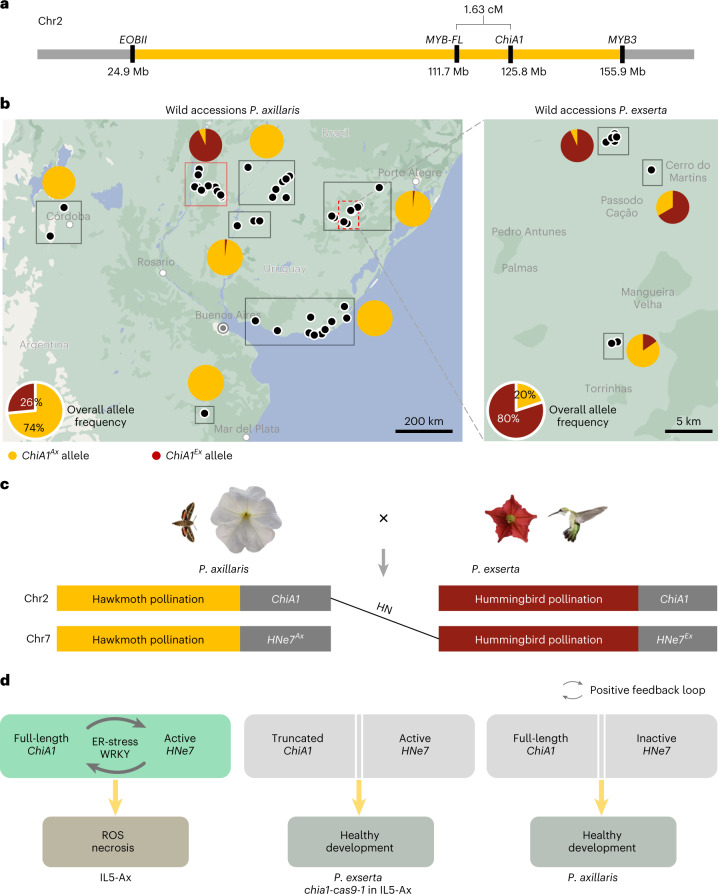
Tight genetic linkage of *ChiA1* with the pollination syndrome genes and the widespread presence of *ChiA1*^*Ax*^ and *ChiA1*^*Ex*^ in nature. **a**, A schematic representation showing the supergene region (yellow coloured) in chromosome 2 between *P. axillaris* and *P. exserta*. The supergene region is defined from *EOBII* to *MYB3* as previously reported by Hermann et al.^19^. **b**, Maps of regions in South America showing the sites (black points) where wild accessions of *P. axillaris* (left) and *P. exserta* (right) were collected. Accessions in a nearby region were grouped for allele frequency calculation (black or red solid squares). Regional allele frequencies are shown as mini pie charts near the squares. Overall allele frequencies for *ChiA1*^*Ax*^ and *ChiA1*^*Ex*^ in the two collections are shown at the lower left corners. Yellow colour indicates *ChiA1*^*Ax*^ allele. Red colour indicates *ChiA1*^*Ex*^ allele. Accessions near the Corrientes region are boxed in red. Red-dashed square comprises the *P. exserta* habitat, shown enlarged on the right. For detailed genotypes and location information, see Supplementary Tables 9 and 10. **c**, Schematic showing the predicted interaction of pre-zygotic and post-zygotic barriers between *P. axillaris* and *P. exserta*. *ChiA1* is tightly linked with several genes controlling pollination syndromes in the supergene. The different pollination syndromes helped the species to adapt to their correspondent pollinators. The HN helps to fix the right combinations of floral traits for its pollinator and thus enhances isolation. **d**, Model of the molecular mechanism of HN. Left: interaction of full-length *P. axillaris* ChiA1 and active *P. exserta* HNe7 induces necrosis. This interaction proceeds through a positive feedback loop involving ER stress and WRKY transcription factor(s). Middle: truncated *P. exserta* or *chia1-cas9-1* ChiA1 fails to induce active *P. exserta* HNe7 because *ChiA1* mRNA does not accumulate due to NMD. Right: full-length *P. axillaris* ChiA1 does not cause symptoms in combination with inactive *P. exserta* HNe7.

*ChiA1* is located at the edge of a region of chromosome 2, where recombination in crosses between *P. axillaris* N and *P. exserta* is strongly reduced^19^ (Fig. 6a). Indeed, *ChiA1* is tightly linked (1.63 cM) to *MYB-FL*, a well-characterized gene inside the supergene that is responsible for the gain and loss of floral UV absorption during evolutionary shifts in pollinator preference^21^ (Fig. 6a and Supplementary Table 7).

**Extended Data Fig. 8:**
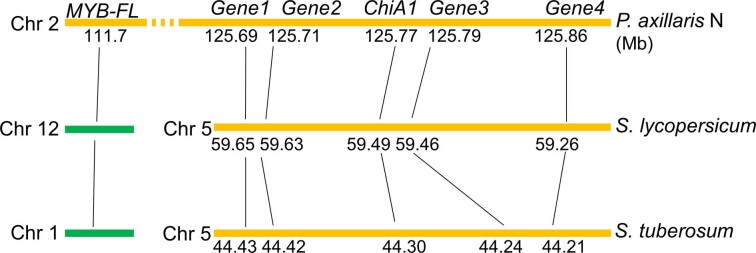
Microsynteny of *ChiA1* with its nearby genes and physical coordinates of *ChiA1* and *MYB-FL* in *P. axillaris* N, *S. lycopersicum* and *S. tuberosum*. The genomic sequences of *ChiA1* and its four nearby genes, as well as *MYB-FL* gene were used for a BLAST search against *P. axillaris* N, *S. lycopersicum*, and *S. tuberosum* genomes. Detailed gene accession numbers, coordinates, and genome versions can be found in Supplementary Table 8 and Methods section.

Analysis of *ChiA1* and its neighbouring genes showed clear microsynteny between *Petunia* and the related Solanaceae species *Solanum lycopersicum* (tomato) and *S. tuberosum* (potato). However, in both these *Solanum* species, *MYB-FL* is not linked to *ChiA1* but resides on a different chromosome (Extended Data Fig. 8 and Supplementary Table 8). The exclusive genetic linkage between *ChiA1* and *MYB-FL* in *Petunia* is consistent with the notion that the ChiA1-mediated HN constitutes a post-zygotic barrier that acts in concert with the pre-zygotic barrier to limit gene flow.

### The *ChiA1*^*Ex*^ allele is widespread in nature

**Extended Data Fig. 9:**
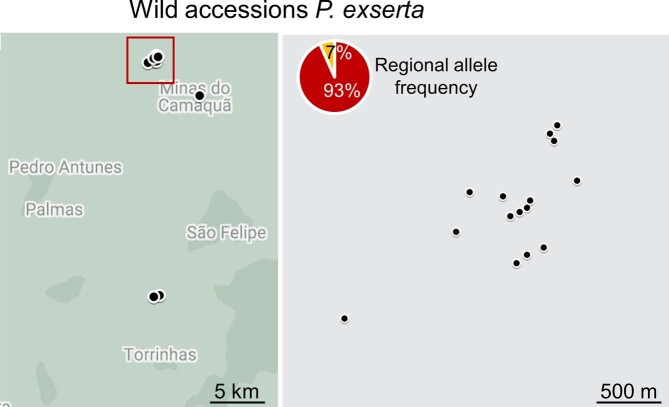
Allele frequency of *ChiA1* in the *P. exserta* wild accessions with a higher resolution. Red squared region was zoomed in in the right panel. Its regional allele frequency is shown as a pie chart on the top left corner. Yellow color indicates *ChiA1*^*Ax*^ allele. Red color indicates *ChiA1*^*Ex*^ allele. For detailed genotypes and location information, see Supplementary Table 9.

To analyse the frequencies of the two alleles of *ChiA1*, we genotyped *ChiA1* across the range of the two species (for details, see Supplementary Tables 9 and 10). *P. exserta* was sampled from 18 sites representing its highly restricted distribution in the Guaritas Region of Brazil. Of the 75 *P. exserta* accessions genotyped, 58 were homozygous for the nonsense mutation (*ChiA1*^*Ex*^), 13 were homozygous for the *P. axillaris* genotype (*ChiA1*^*Ax*^) and 4 were heterozygous (Fig. 6b, right, Extended Data Fig. 9 and Supplementary Table 9). The overall *ChiA1*^*Ex*^ allele frequency among these accessions was 80% (Fig. 6b, right).

*P. axillaris* accessions were sampled from multiple locations in Argentina, Uruguay and Brazil, including the regions of sympatry with *P. exserta* (Fig. 6b, left, red-dashed square). Of the 238 *P. axillaris* accessions, 173 were homozygous for the *P. axillaris* allele (*ChiA1*^*Ax*^), 61 were homozygous for the *P. exserta* allele (*ChiA1*^*Ex*^) and 4 were heterozygous (Fig. 6b, left, and Supplementary Table 10). The overall *ChiA1*^*Ax*^ allele frequency among these accessions was 74% (Fig. 6b, left). Sixty-two *P. axillaris* accessions with the *ChiA1*^*Ex*^ allele are located in the Corrientes and Entre Ríos regions in Argentina (Fig. 6b, solid square in red), which is ~500 km away from the Guaritas Region where *P. exserta* accessions are exclusively found. These accessions belong to *P. axillaris* subspecies *parodii*^48^, which is distinguished from *P. axillaris* ssp. *axillaris* mainly by its longer corolla tube^49^.

These results confirm the presence of both *ChiA1* alleles in the natural *P. axillaris* and *P. exserta* populations. *ChiA1*^*Ex*^ is more present in *P. exserta*, whereas *ChiA1*^*Ax*^ is more frequent in *P. axillaris*.

## Discussion

We discovered a case of HN in crosses between *P. axillaris* and *P. exserta* relying on a deleterious epistatic interaction between the *HNe2* and *HNe7* loci (Fig. 6c). The gene underlying *HNe2* is *ChiA1*, which encodes a protein with both chitinase and lysozyme activity. *ChiA1*^*Ax*^ allele encodes a functional enzyme, whereas *ChiA1*^*Ex*^ has a nonsense mutation leading to a premature stop codon.

Cases of HN characterized in multiple species often have commonalities: activation of immune responses and the direct participation of immune system components^10^. In a well-studied case in tomato, a secreted cysteine protease causes autoimmunity when the allele from a related wild species is present in combination with an avirulence (Avr) receptor of the cultivated tomato^50,51^. In this example, however, the protease activity of the enzyme is thought to be essential, whereas replacement of the critical amino acids in the active site of ChiA1 does not interfere with its ability to cause necrosis (Fig. 4e).

If it is not the activity of the protein, by what mechanism does *ChiA1* cause necrosis? The level of expression is high for *ChiA1*^*Ax*^ in IL5-Ax but low for the *ChiA1*^*Ex*^ and *chia1-cas9-1* alleles. The low expression of *ChiA1*^*Ex*^ and *chia1-cas9-1* is mostly probably caused by the mRNA surveillance pathway through the mRNA NMD mechanisms^38^. In contrast, *ChiA1*^*Ax*^ is one of the most highly expressed genes in the necrotic IL5-Ax background. The high expression of ER stress marker genes in the same background indicates ER stress. Many key pathogenesis-related proteins rely on ER and ER quality control for proper folding and secretion^52–54^. We propose that the high and chronic demand for the processing of ChiA1 in the ER exceeds the ER quality control working capacity and causes prolonged ER stress and cell death^55–57^.

Considering the extremely high expression of ChiA1^Ax^ in the necrotic line, a positive feedback loop between the two interacting loci seems plausible (Fig. 6d). The basal expression of *ChiA1*^*Ax*^ in the necrotic line may trigger the activation of *HNe7*^*E*x^ (probably via WRKY18), and thus HNe7^Ex^ induces autoimmunity, which further enhances the expression of *ChiA1*. In this case, *HNe7* may encode an auto-active immune system component (for example, NLR^10^). Alternatively, HNe7^Ex^ activates the expression of *ChiA1*^*Ax*^, and ChiA1^Ax^ induces the autoimmunity. In that case, HNe7 might be a transcriptional or post-transcriptional regulator. *HNe7* is located in a large region of very low recombination containing close to 1,000 genes, and its identity is not yet known. Thus, the molecular details of the interaction of ChiA1 and HNe7 remain to be determined.

A unique aspect of the work presented here is that *ChiA1* is tightly linked to a region of low recombination containing major genes involved in the evolution of pollination syndromes. Among them is *MYB-FL*, encoding a transcription factor that induces the synthesis of UV-absorbing pigments in the moth-pollinated *P. axillaris* but was inactivated in the hummingbird pollinated *P. exserta*^19,21^. This genetic linkage is relatively recent, as *ChiA1* and *MYB-FL* are located on different chromosomes in tomato and potato (Extended Data Fig. 8). This association may have served to enhance reproductive isolation during the process of speciation.

Both the *ChiA1*^*Ax*^ and *ChiA1*^*Ex*^ alleles were found in *P. axillaris* as well as in *P. exserta* wild accessions collected from multiple sites in South America (Fig. 6b). The presence of *ChiA1*^*Ax*^ in wild *P. exserta* could be due to introgression^58^. *P. axillaris* ssp*. parodii* does not reach the *P. exserta* region today, and in the past, there were no conditions suitable for *P. parodii* in Serra do Sudeste, even considering Last Glacial Maximum (~ 22 kya) or mid-Holocene (6 kya)^59^. The presence of *ChiA1*^*Ex*^ in *P. axillaris* ssp*. parodii* strongly suggests incomplete lineage sorting^60^, consistent with a function in reproductive isolation during speciation. As chitinases are important in pattern-triggered immunity against pathogens, the loss of ChiA1 function is likely to compromise defence. The potential benefit of the *ChiA1*^*Ex*^ allele in allopatric *P. axillaris* ssp*. parodii* populations will need further study. The presence of *ChiA1*^*Ax*^ in *P. exserta* suggests conditions in which the benefits of pathogen resistance outweigh its effect on reproductive isolation.

Speciation occurs when reproductive barriers accumulate and substantially reduce gene flow between lineages^3^. Pollinator preference is a strong barrier against gene flow, but it is rarely absolute^61^. This is also true for *Petunia*^62^. Multiple reproductive barriers acting in concert are therefore often needed to complete reproductive isolation^63,64^. The genetic linkage of distinct isolation mechanisms would further enhance reproductive isolation and thereby enhance the tempo of diversification and speciation. Such linkage may be a more general phenomenon that can help explain the rapid and successful diversification of the angiosperms.

## Methods

### Plant materials

*P. axillaris* N is from the Rostock Botanical Garden (Germany), *P. exserta* is from R.J. Griesbach (Beltsville, United States). The accessions are maintained by self-fertilization. The introgression line IL5 has been described previously^28^. Wild accessions of *P. axillaris* and *P. exserta* have been described previously^21,23,25,48^ and are described further in Supplementary Tables 9 and 10.

Plants were grown in a greenhouse or a growth chamber. Greenhouse plants were grown with additional lighting resulting in 14 h day at 18–25 °C in pots. Plants grown in a growth chamber are under a light:dark regime of 15 h:9 h, at 22 °C:17 °C at 60%–80% relative humidity, in commercial soil (70% Klasman substrate, 15% Seramis clay granules and 15% quartz sand) and fertilized once a week (Plantaktiv, 16+6+26 type K fertilizer, 0.1% concentration).

### DAB staining

Diaminobenzidine (DAB) staining was performed according to a previous protocol^65^. After the treatment, the leaf was used for immediate observation or kept at 4 °C.

### RNA extraction and quantitative PCR

Total RNA was extracted from leaf tissues using an innuPREP DNA/RNA Mini Kit (Analytik Jena; code 845-KS-20800250). Complementary DNA was synthesized using qScriber cDNA Synthesis Kit (HighQu; code RTK0104). Quantitative PCR reactions were set up with ORA SEE qPCR Green ROX L Mix (HighQu; code QPD0505). The amplification was performed using a QuantStudio 5 Real-Time PCR Instrument (Applied Biosystems). Leaves were sampled from three individual plants for each genotype. We took the seventh-oldest leaves (counting from bottom to up; approximately sixth-newest counting from up to bottom) from the 10-week-old plants as this leaf in IL5-Ax plants starts to show necrotic symptoms at this stage. The data were analysed by the ∆∆Ct method^66^, and normalized by the housekeeping *RAN1* gene^21^. Primer pairs for quantitative PCR are listed in Supplementary Table 11.

### Leaf yellowness analysis

**Extended Data Fig. 10:**
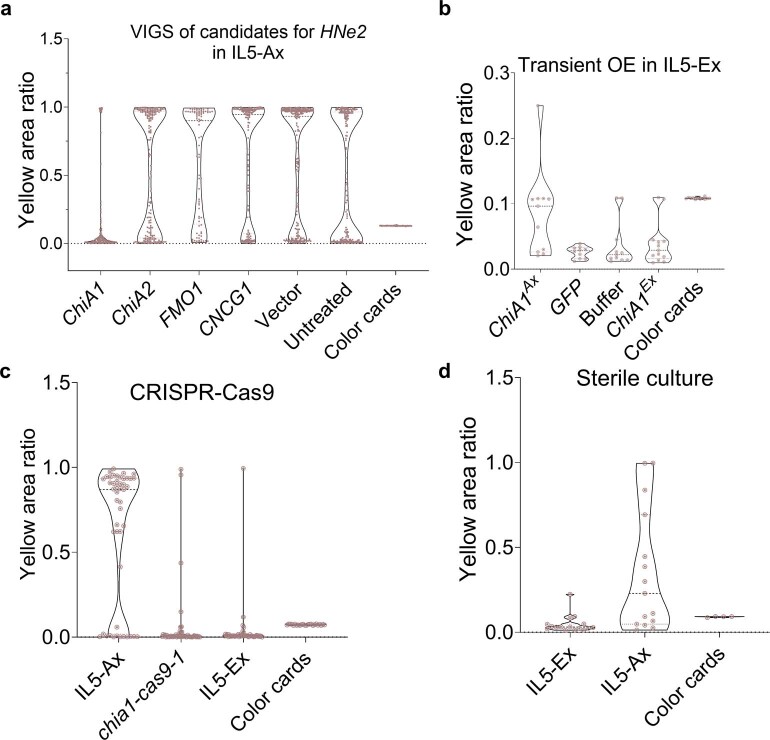
Consistency of picture light condition. The consistency of the yellow areas identified through each set of pictures from Fig. 3c (a), 3e (b), 3h (c), 4d (d) was validated using the color card included in each picture.

The ratio of leaf yellow area was calculated on the basis of a previous method with modifications^67^. Briefly, leaves from each plant were removed and photographed on a black background under uniform artificial light conditions with a colour reference card included in each photo. The software Fiji (ImageJ) was used to process the pictures^68^. Total leaf area as well as yellow and green leaf area were measured on the basis of colour thresholds for hue, saturation and brightness values. Only pictures taken in the same session were compared to guarantee light uniformity. To define the hue parameters that identify a region as yellow, we first applied the parameters to the colour reference card in each photo and verified that the yellow areas identified were consistent. The yellow area measured on the colour cards is shown in Extended Data Fig. 10. We then used the hue thresholds to analyse the leaf photos. The Fiji macro outputs a painted version of the photos, where the yellow area measured is shown. We manually verified that this area correctly overlapped the yellow parts of the leaves. Detailed scripts and instructions can be found here: https://github.com/Kuhlemeier-lab/fiji_macros.

### BSR-seq and data analysis

We used the method for BSR-seq (a bulk-segregant analysis based on RNA-seq data) published by Soyk et al.^69^ to map the loci responsible for HN. *P. axillaris* N (mother plant) and *P. exserta* (pollen donor) were used to produce an F2 population. A total of 384 F2 plants were sown for sampling. On the basis of the phenotypic scale shown in Fig. 2a, we selected plants 13 weeks after sowing showing a strong necrotic leaf phenotype (19 individuals, score 4) or showing a healthy leaf phenotype (89 individuals, score 0). A leaf disc (Ø = 9 mm) from a mature and healthy leaf of each plant was collected, flash frozen in liquid nitrogen and stored at −80 °C. Total RNA was extracted from pools of leaf discs either from the necrotic plants or the healthy plants. RNA-seq was performed by NovoGene with a polyA-enriched library prep, to obtain paired-end reads of 150 bp. Alignment of quality-controlled reads was performed with STAR^70^ (2.6.0c) on the reference genome of *P. axillaris* N version 4.03. Variants were called using GATK^71^ (4.0.4.0). The variants were filtered to keep only high-quality, biallelic single-nucleotide polymorphisms (SNPs) with a minimum read depth of 100 in each sample (using minDP argument in vcftools), and with minimum 100 bp between each variant position. We calculated the difference in alternate allele frequency between the two pools (necrotic - healthy) and used this ∆SNP frequency to define genome-wide thresholds at 0.01 and 0.05 (lower thresholds) and 0.95 and 0.99 (higher thresholds) quantiles. The SNPs were then grouped in stepping windows of 100 SNPs along the genome and the proportion of SNPs with value outside of the genome-wide thresholds in each window was used to produce the main figure. Allele frequency calculations and plotting was performed in R^72^. Detailed parameters, software versions and scripts are deposited on Github: https://github.com/Kuhlemeier-lab/Petunia_hybrid_necrosis#bsr-seq.

### Fine mapping using recombinant lines of IL5-Het

As IL5-Het is a near-isogenic line segregating for a chromosome 2 region comprising *HNe2* while the rest of the genome is homozygous, we use it as a starting material to search for recombinant lines among its progenies and reduce the size of the interval of interest. We screened 3,800 progenies of IL5-Het with the markers mHIND-cn9140 and mHIND-MYB58 that are flanking the heterozygous region, allowing the identification of 37 recombinant lines (Supplementary Table 1). These lines harbour a smaller heterozygous region compared with IL5-Het that were genotypically characterized with the addition of more genetic markers (Supplementary Tables 1 and 11). Then, phenotypic characterization of homozygous progenies of these recombinant lines allowed the fine mapping of *HNe2* down to an 8.7 Mb region (Supplementary Table 1).

To get a better resolution of the recombination breakpoints and reduce the *HNe2* locus, ten informative recombinant lines and four controls homozygous for the *HNe2* region (two *P. axillaris* and two *P. exserta*) were selected for whole genome sequencing (Supplementary Table 1). Genomic DNA extraction from leaf tissue of the selected lines was performed with a modified CTAB method as previously reported^73^. DNA was sequenced by the Next Generation Sequencing platform of the University of Bern using Illumina whole genome sequencing library preparation to obtain paired-end 150 bp reads. Quality-controlled reads were aligned with BWA MEM default parameters^74^ to the genome of *P. axillaris* N v. 4.03, providing an average coverage of 3.3 × along the genome. Variants were called and quality filtered with GATK^71^. Observation of the genotypes of the variants associated with the HN phenotype in the recombinant lines allowed reduction of the size of the region containing *HNe2*. The detailed software versions, parameters and scripts are deposited on Github: https://github.com/Kuhlemeier-lab/Petunia_hybrid_necrosis#il-shallow-sequencing.

### RNA-seq analysis

IL5-Ax and IL5-Ex plants coming from the same IL5-Het parent were grown for leaf sampling. The leaf displaying the onset of necrosis in IL5-Ax was harvested, and equivalent leaves were sampled for the other genotypes. Three biological replicates from three different plants were set for each genotype. Total RNA was extracted from leaf tissues using an innuPREP DNA/RNA Mini Kit (Analytik Jena; code: 845-KS-20800250). Quality controls were performed using a Nanodrop (NanoDrop 1000, Thermo Fisher) and a fragment analyser (2100 Bioanalyzer Instrument, Agilent). RNA-seq was performed by Lausanne Genomic Technologies Facility (University of Lausanne) with TruSeq stranded RNA library prep to obtain single-end reads of 125 bp. Quality-controlled reads were aligned to the reference genome of *P. axillaris* version 4.03 using STAR^70^. Read counts were generated with Subread^75^. Differential expression analysis was performed with DESeq2 in R^76^ between IL5-Ax and IL5-Ex (*q* < 0.001). The detailed software versions, parameters and scripts are deposited on Github: https://github.com/Kuhlemeier-lab/Petunia_hybrid_necrosis#rnaseq.

### Phylogenetic analysis

ChiA1 and its homologous sequences from species listed in Extended Data Fig. 4c were identified in the NCBI nr (nonredundant protein sequences) database by performing a BLASTp search with ChiA1 protein sequences (Supplementary Table 12). Amino acid sequences were aligned with MUSCLE in the MEGA-X software package using the default settings for protein multiple alignments. Evolutionary distances were computed using Poisson correction analysis. The bootstrap method with 1,000 replicates for phylogeny testing was used.

### Protein structure prediction

The structures of ChiA1 variants were predicted by AlphaFold^77^. The structures were visualized and aligned by PyMOL (Version 2.5.3, Schrödinger LLC).

### VIGS

VIGS was performed as described previously^23^. For each candidate gene, we targeted a specific part of the coding region to avoid off-targeting (Supplementary Table 11). After amplification with primers containing BamHI and EcoRI restriction sites, these fragments were cloned into the pTRV2-MCS plasmid (ABRC accessions CD3-1040) and transformed into *Agrobacterium tumefaciens* strain GV3101. Seven weeks after the infection, only leaves from the branches arising from infected meristems were phenotyped.

### Transient and stable overexpression

*ChiA1*^*D148A*, *E150A*^ coding sequence was generated by PCR mutagenesis method with *ChiA1*^*Ax*^ as a template. The coding sequences of *ChiA1*^*Ax*^, *ChiA1*^*D148A*, *E150A*^ and *ChiA1*^*Ex*^ were amplified with primers containing AttB sites (Supplementary Table 11) and cloned into the Gateway-compatible binary vector pGWB402 (Addgene plasmid #74796) containing a CaMV35S promoter. For transient overexpression, the constructs were transformed into *Agrobacterium tumefaciens* strain GV3101. The *Agrobacterium* cells were grown to OD_600_ of 0.8, pelleted, and resuspended in infiltration buffer (10 mM methylester sulfonate, 10 mM MgCl_2_ and 150 µM acetosyringone, pH 5.7) and infiltrated into 7- or 8-week-old *P. exserta* or 5-week-old *Nicotiana benthamiana* plant leaves using a needleless syringe. The phenotype was observed 2 weeks (*Petunia*) or 5 days (*Nicotiana*) after the infiltration. Stable transgenic *P. axillaris* N lines were generated by leaf disc transformation with *Agrobacterium tumefaciens* strain GV3101 following an adapted protocol^23^ based on Conner et al.^78^.

### CRISPR–Cas9 editing

CRISPR–Cas9 construct for *ChiA1* pHSE401(Neo/Kana)-U6-26p > CHIAb[gRNA#1]-U6-26p>CHIAb[gRNA#2] was ordered from VectorBuilder (https://en.vectorbuilder.com/). gRNA 1: CTCACGTCCACTAGGAGATG, gRNA 2: AGGGAGGGACAGCAGAACAT. The construct was transformed into *Agrobacterium tumefaciens* strain LBA4404. IL5-Ax CRISPR–Cas9 line was generated with the *Agrobacterium* by leaf disc transformation following the same protocol for stable transgenic *P. axillaris* N lines. Editing in the T0 generation was detected by PCR performed on genomic DNA with primers targeting the gRNA sites (Supplementary Table 11) followed by Sanger sequencing.

### Protein extraction, chitinase and lysozyme activity measurement

Leaf apoplastic proteins were obtained by vacuum infiltration of 7-week-old *Petunia* plant leaves (the stage when IL5-Ax plants start to show necrotic symptoms) with activity buffer from Chitinase Assay Kit (abbexa, catalogue: abx298854) or Lysozyme Activity Assay Kit (Fluorometric) (Abcam, catalogue: ab211113). The leaves were dried on a paper towel. Then leaf discs (Ø = 9 mm) were obtained by a hole puncher so that the total area of leaves used for protein extraction could be determined. Twenty-two leaf discs from three plants were pooled as one biological replicate. Afterward, the pooled leaf discs were placed in a 50 ml Falcon tube and spun at 1,000 g for 5 min at 4 °C. Three biological replicates (3 × 22 leaf discs from nine plants) were set for each genotype tested.

Chitinase activity was detected with a Chitinase Assay Kit (abbexa, code: abx298854) following its standard protocol. One unit of chitinase activity was defined as the amount of enzyme required to produce 1 µg of *N*-acetylglucosamine per hour at 37 °C. Lysozyme activity was detected with Lysozyme Activity Assay Kit (Fluorometric) (Abcam, code: ab211113) following its standard protocol. One unit of lysozyme activity was defined as the amount of enzyme that generates 1.0 μmol of 4-MU per minute at pH 5.0 at 37 °C.

### Sterile culture

Transparent 5-litre beakers were used as containers for the sterile culture. The beakers with 1.5 litre commercial soil (70% Klasman substrate, 15% Seramis clay granules and 15% quartz sand) were sealed with aluminium foil and autoclaved at 121 °C for 20 min. Then 500 ml autoclaved water was added to the beaker in a sterile environment under laminar flow. Seeds with the genotype of IL5-Ax and IL5-Ex were sterilized with 1% bleach for 10 min and rinsed in sterile water five times. Then the seeds were sowed in the beakers in a sterile environment. The beakers were sealed with several layers of transparent plastic film and put in the growth chamber. The phenotypes of the leaves were analysed 12 weeks after sowing. Four biological replicates for each genotype were observed with similar results.

### Conserved *cis*-elements screening

One-kilobase promoter sequences (1 kb upstream of translation start codons) of the DEGs were extracted and subjected to the motif discovery by MEME-ChIP^79^ (http://meme-suite.org/tools/meme-chip) with default parameters.

### Genotyping

Genotypes of *ChiA1* in the wild accessions of *P. axillaris* and *P. exserta* were determined by the CAPS marker designed on the nonsense mutation site. The G-to-T mutation in *P. exserta* allele of *ChiA1* impairs the FokI (GGATGN7) restriction enzyme digestion site. CAPS marker primer sequences can be found in Supplementary Table 11. Genotypes of *MYB-FL* were determined as previously described^21^.

### Microsynteny analysis

Sequences of *MYB-FL*, *ChiA1* and its four nearby genes in the genome were BLASTed against the ITAG release 4.0 cDNA library for *S. lycopersicum* and PGSC DM4.03 cDNA library for *S. tuberosum*. For detailed gene IDs and coordinates of the genes shown in the microsynteny analysis, see Supplementary Table 8.

### Statistical analysis

GraphPad Prism v.6.0.7 and Microsoft Excel 2016 were used for the statistical analyses (one-way analysis of variance (ANOVA) and two-sided Student’s *t*-test).

### Reporting summary

Further information on research design is available in the Nature Portfolio Reporting Summary linked to this article.

## Supplementary information

Reporting Summary

Supplementary Table 1 Supplementary Table 1. Fine mapping of *HNe2* using recombinant lines derived from IL5-Het. Supplementary Table 2. List of genes inside the final *HNe2* region. Supplementary Table 3. Expression level (normalized read counts) of the genes present in the *HNe2* region. Supplementary Table 4. Features of the 34 expressed genes present in the *HNe2* region. Supplementary Table 5. List of all the genes differentially expressed between IL5-Ax and IL5-Ex plant leaves. Supplementary Table 6. List of DEGs between IL5-Ax and IL5-Ex leaves that carry a WRKY binding site(s) shown in Fig. 5a in the promoter regions. Supplementary Table 7. Genetic distance between *ChiA1* and *MYB-FL* analysed by an F2 population of 369 individuals. Supplementary Table 8. Gene ID and coordinates of the genes used in the microsynteny analysis. Supplementary Table 9. Detailed information of individuals in the *P. exserta* wild accessions. Supplementary Table 10. Detailed information of individuals in the *P. axillaris* wild accessions. Supplementary Table 11. Primer list. Supplementary Table 12. Protein IDs and sequences used in phylogenetic analysis of ChiA1.

## Data Availability

BSR-seq reads have been deposited in the NCBI Sequence Read Archive (SRA) under BioProject PRJNA708139. Shallow whole-genome sequencing reads data have been deposited under BioProject PRJNA705072. RNA-seq reads have been deposited under BioProject PRJNA705649. The *P. axillaris* N 4.03 genome assembly has been deposited at NCBI GenBank under the accession JANRMM000000000 (https://www.ncbi.nlm.nih.gov/bioproject/?term=JANRMM000000000).

## Source data

Source Data Fig. 1 Statistical source data.
Source Data Fig. 3 Statistical source data.
Source Data Fig. 4 Statistical source data.
Source Data Fig. 5 Statistical source data.
Source Data Extended Data Fig. 1 Statistical source data.
Source Data Extended Data Fig. 4 Statistical source data.
Source Data Extended Data Fig. 7 Statistical source data.

## Code availability

All scripts used in this paper have been deposited on Github: https://github.com/Kuhlemeier-lab/Petunia_hybrid_necrosis. Major computations were performed on a SLURM computing cluster (UBELIX http://www.id.unibe.ch/hpc, the HPC cluster at the University of Bern).

## References

[1] Coyne JA, Orr HA (1998). The evolutionary genetics of speciation. Philos. Trans. R. Soc. Lond. B.

[2] Coyne, J.A. & Orr, H.A. *Speciation*, xiii, 545, 2 p. of plates (Sinauer Associates, 2004).

[3] Rieseberg LH, Willis JH (2007). Plant speciation. Science.

[4] Grant, V. *Plant Speciation* (Columbia Univ. Press, 1981).

[5] Fenster CB, Armbruster WS, Wilson P, Dudash MR, Thomson JD (2004). Pollination syndromes and floral specialization. Annu. Rev. Ecol. Evol. Syst..

[6] Alcazar R, Garcia AV, Parker JE, Reymond M (2009). Incremental steps toward incompatibility revealed by *Arabidopsis* epistatic interactions modulating salicylic acid pathway activation. Proc. Natl Acad. Sci. USA.

[7] Bomblies K, Weigel D (2007). Hybrid necrosis: autoimmunity as a potential gene-flow barrier in plant species. Nat. Rev. Genet..

[8] Seehausen O (2014). Genomics and the origin of species. Nat. Rev. Genet..

[9] Orr HA, Turelli M (2001). The evolution of postzygotic isolation: accumulating Dobzhansky–Muller incompatibilities. Evolution.

[10] Li L, Weigel D (2021). One hundred years of hybrid necrosis: hybrid autoimmunity as a window into the mechanisms and evolution of plant–pathogen interactions. Annu Rev. Phytopathol..

[11] Chae E (2014). Species-wide genetic incompatibility analysis identifies immune genes as hot spots of deleterious epistasis. Cell.

[12] Bomblies K (2007). Autoimmune response as a mechanism for a Dobzhansky–Muller-type incompatibility syndrome in plants. PLoS Biol..

[13] Barragan AC (2021). A truncated singleton NLR causes hybrid necrosis in *Arabidopsis thaliana*. Mol. Biol. Evol..

[14] Maekawa T, Kufer TA, Schulze-Lefert P (2011). NLR functions in plant and animal immune systems: so far and yet so close. Nat. Immunol..

[15] Stehmann, J.R., Lorenz-Lemke, A.P., Freitas, L.B. & Semir, J. in *Petunia: Evolutionary, Developmental and Physiological Genetics* (eds Gerats, T. & Strommer, J.) 1–28 (Springer New York, 2009).

[16] Galliot C, Stuurman J, Kuhlemeier C (2006). The genetic dissection of floral pollination syndromes. Curr. Opin. Plant Biol..

[17] Venail J, Dell’olivo A, Kuhlemeier C (2010). Speciation genes in the genus *Petunia*. Philos. Trans. R. Soc. Lond. B.

[18] Lorenz-Lemke AP (2006). Diversity and natural hybridization in a highly endemic species of *Petunia* (Solanaceae): a molecular and ecological analysis. Mol. Ecol..

[19] Hermann K (2013). Tight genetic linkage of prezygotic barrier loci creates a multifunctional speciation island in *Petunia*. Curr. Biol..

[20] Amrad A (2016). Gain and loss of floral scent production through changes in structural genes during pollinator-mediated speciation. Curr. Biol..

[21] Sheehan H (2016). MYB-FL controls gain and loss of floral UV absorbance, a key trait affecting pollinator preference and reproductive isolation. Nat. Genet..

[22] Yarahmadov T, Robinson S, Hanemian M, Pulver V, Kuhlemeier C (2020). Identification of transcription factors controlling floral morphology in wild *Petunia* species with contrasting pollination syndromes. Plant J..

[23] Berardi AE (2021). Complex evolution of novel red floral color in *Petunia*. Plant Cell.

[24] Reck-Kortmann M (2014). Multilocus phylogeny reconstruction: new insights into the evolutionary history of the genus *Petunia*. Mol. Phylogenet. Evol..

[25] Segatto AL (2014). Nuclear and plastid markers reveal the persistence of genetic identity: a new perspective on the evolutionary history of *Petunia exserta*. Mol. Phylogenet. Evol..

[26] Schnitzler CK, Turchetto C, Teixeira MC, Freitas LB (2020). What could be the fate of secondary contact zones between closely related plant species?. Genet. Mol. Biol..

[27] Liu X (2014). Host-induced bacterial cell wall decomposition mediates pattern-triggered immunity in *Arabidopsis*. eLife.

[28] Hermann K, Klahre U, Venail J, Brandenburg A, Kuhlemeier C (2015). The genetics of reproductive organ morphology in two Petunia species with contrasting pollination syndromes. Planta.

[29] Iwata Y, Fedoroff NV, Koizumi N (2008). *Arabidopsis* bZIP60 is a proteolysis-activated transcription factor involved in the endoplasmic reticulum stress response. Plant Cell.

[30] Howell SH (2013). Endoplasmic reticulum stress responses in plants. Annu. Rev. Plant Biol..

[31] Lee HY (2008). Incompatibility of nuclear and mitochondrial genomes causes hybrid sterility between two yeast species. Cell.

[32] Meiklejohn CD (2013). An Incompatibility between a mitochondrial tRNA and its nuclear-encoded tRNA synthetase compromises development and fitness in *Drosophila*. PLoS Genet..

[33] Lin W (1995). Genetic-engineering of rice for resistance to sheath blight. Nat. Biotechnol..

[34] Chen YC (2018). *N*-hydroxy-pipecolic acid is a mobile metabolite that induces systemic disease resistance in *Arabidopsis*. Proc. Natl Acad. Sci. USA.

[35] Ma W, Smigel A, Verma R, Berkowitz GA (2009). Cyclic nucleotide gated channels and related signaling components in plant innate immunity. Plant Signal Behav..

[36] Balague C (2003). HLM1, an essential signaling component in the hypersensitive response, is a member of the cyclic nucleotide-gated channel ion channel family. Plant Cell.

[37] Lu R, Martin-Hernandez AM, Peart JR, Malcuit I, Baulcombe DC (2003). Virus-induced gene silencing in plants. Methods.

[38] Chang YF, Imam JS, Wilkinson MF (2007). The nonsense-mediated decay RNA surveillance pathway. Annu. Rev. Biochem..

[39] Jekel PA, Hartmann BH, Beintema JJ (1991). The primary structure of hevamine, an enzyme with lysozyme/chitinase activity from Hevea brasiliensis latex. Eur. J. Biochem..

[40] Yu X, Feng B, He P, Shan L (2017). From chaos to harmony: responses and signaling upon microbial pattern recognition. Annu. Rev. Phytopathol..

[41] van Aalten DM (2001). Structural insights into the catalytic mechanism of a family 18 exo-chitinase. Proc. Natl Acad. Sci. USA.

[42] Synstad B (2004). Mutational and computational analysis of the role of conserved residues in the active site of a family 18 chitinase. Eur. J. Biochem..

[43] Malolepszy A (2018). A plant chitinase controls cortical infection thread progression and nitrogen-fixing symbiosis. eLife.

[44] Aerts N, Chhillar H, Ding P, Van Wees SCM (2022). Transcriptional regulation of plant innate immunity. Essays Biochem..

[45] Rushton PJ, Somssich IE, Ringler P, Shen QJ (2010). WRKY transcription factors. Trends Plant Sci..

[46] Tsuda K, Somssich IE (2015). Transcriptional networks in plant immunity. N. Phytol..

[47] Franco-Zorrilla JM (2014). DNA-binding specificities of plant transcription factors and their potential to define target genes. Proc. Natl Acad. Sci. USA.

[48] Turchetto C (2014). Diversification in the South American Pampas: the genetic and morphological variation of the widespread *Petunia axillaris* complex (Solanaceae). Mol. Ecol..

[49] Steere WC (1931). *Petunia parodii*, a new species of the subgenus *Pseudonicotiana* from Argentina. J. Pap. Mich. Acad. Sci. Arts Lett..

[50] Kruger J (2002). A tomato cysteine protease required for Cf-2-dependent disease resistance and suppression of autonecrosis. Science.

[51] Dixon MS, Golstein C, Thomas CM, van Der Biezen EA, Jones JD (2000). Genetic complexity of pathogen perception by plants: the example of Rcr3, a tomato gene required specifically by Cf-2. Proc. Natl Acad. Sci. USA.

[52] Li J (2009). Specific ER quality control components required for biogenesis of the plant innate immune receptor EFR. Proc. Natl Acad. Sci. USA.

[53] Wang D, Weaver ND, Kesarwani M, Dong X (2005). Induction of protein secretory pathway is required for systemic acquired resistance. Science.

[54] Simoni EB, Oliveira CC, Fraga OT, Reis PAB, Fontes EPB (2022). Cell death signaling from endoplasmic reticulum stress: plant-specific and conserved features. Front. Plant Sci..

[55] Kang YW, Jeon Y, Pai HS (2012). Characterization of cell death induced by NbBPS1 silencing in *Nicotiana benthamiana*. Mol. Cells.

[56] Liu JX, Howell SH (2016). Managing the protein folding demands in the endoplasmic reticulum of plants. N. Phytol..

[57] Moon JY, Lee JH, Oh CS, Kang HG, Park JM (2016). Endoplasmic reticulum stress responses function in the HRT-mediated hypersensitive response in *Nicotiana benthamiana*. Mol. Plant Pathol..

[58] Turchetto C (2019). Contact zones and their consequences: hybridization between two ecologically isolated wild *Petunia* species. Bot. J. Linn. Soc..

[59] Giudicelli GC, Turchetto C, Silva-Arias GA, Freitas LB (2019). Influence of climate changes on the potential distribution of a widespread grassland species in South America. Perspect. Plant Ecol. Evol. Syst..

[60] Mailund T, Munch K, Schierup MH (2014). Lineage sorting in apes. Annu Rev. Genet..

[61] Baack E, Melo MC, Rieseberg LH, Ortiz-Barrientos D (2015). The origins of reproductive isolation in plants. N. Phytol..

[62] Brandenburg A, Kuhlemeier C, Bshary R (2012). Hawkmoth pollinators decrease seed set of a low-nectar *Petunia axillaris* line through reduced probing time. Curr. Biol..

[63] Butlin RK, Smadja CM (2018). Coupling, reinforcement, and speciation. Am. Nat..

[64] Christie K, Strauss SY (2019). Reproductive isolation and the maintenance of species boundaries in two serpentine endemic Jewelflowers. Evolution.

[65] Daudi A, O’Brien JA (2012). Detection of hydrogen peroxide by DAB staining in *Arabidopsis* leaves. Bio Protoc..

[66] Winer J, Jung CK, Shackel I, Williams PM (1999). Development and validation of real-time quantitative reverse transcriptase-polymerase chain reaction for monitoring gene expression in cardiac myocytes in vitro. Anal. Biochem..

[67] Laflamme B, Middleton M, Lo T, Desveaux D, Guttman DS (2016). Image-based quantification of plant immunity and disease. Mol. Plant Microbe Interact..

[68] Schindelin J (2012). Fiji: an open-source platform for biological-image analysis. Nat. Methods.

[69] Soyk S (2017). Variation in the flowering gene SELF PRUNING 5G promotes day-neutrality and early yield in tomato. Nat. Genet..

[70] Dobin A (2013). STAR: ultrafast universal RNA-seq aligner. Bioinformatics.

[71] Van der Auwera, G. A. et al. From FastQ data to high-confidence variant calls: the Genome Analysis Toolkit best practices pipeline. *Curr. Protoc. Bioinform.* **43**, 1–33 (2013). 10.1002/0471250953.bi1110s43 PMC4243306 25431634

[72] R: A language and environment for statistical computing (R Core Team, 2013).

[73] Esfeld K (2018). Pseudogenization and resurrection of a speciation gene. Curr. Biol..

[74] Li H, Durbin R (2009). Fast and accurate short read alignment with Burrows–Wheeler transform. Bioinformatics.

[75] Liao Y, Smyth GK, Shi W (2014). featureCounts: an efficient general purpose program for assigning sequence reads to genomic features. Bioinformatics.

[76] Love MI, Huber W, Anders S (2014). Moderated estimation of fold change and dispersion for RNA-seq data with DESeq2. Genome Biol..

[77] Jumper J (2021). Highly accurate protein structure prediction with AlphaFold. Nature.

[78] Conner, A.J., Albert, N.W. & Deroles, S.C. in *Petunia: Evolutionary, Developmental and Physiological Genetics* (eds Gerats, T. & Strommer, J.) 395–409 (Springer New York, 2009).

[79] Machanick P, Bailey TL (2011). MEME-ChIP: motif analysis of large DNA datasets. Bioinformatics.

