# Supplementary material for: Tight genetic linkage of genes causing hybrid necrosis and pollinator isolation between young species
Source: Nat Plants. 2023 Feb 20;9(3):420–32. doi: 10.1038/s41477-023-01354-8 (PMC10027609; doi:10.1038/s41477-023-01354-8)
Supplement: Supplementary file 1 — Reporting Summary [file 41477_2023_1354_MOESM1_ESM.pdf]

nature portfolio

Corresponding author(s): Cris Kuhlemeier, Mathieu Hanemian

Last updated by author(s): Jan 16, 2023

Reporting Summary

Nature Portfolio wishes to improve the reproducibility of the work that we publish. This form provides structure for consistency and transparency in reporting. For further information on Nature Portfolio policies, see our Editorial Policies and the Editorial Policy Checklist.

Statistics

For all statistical analyses, confirm that the following items are present in the figure legend, table legend, main text, or Methods section.

n/a	Confirmed	
☐	☒	The exact sample size (*n*) for each experimental group/condition, given as a discrete number and unit of measurement
☐	☒	A statement on whether measurements were taken from distinct samples or whether the same sample was measured repeatedly
☐	☒	The statistical test(s) used AND whether they are one- or two-sided<br>*Only common tests should be described solely by name; describe more complex techniques in the Methods section.*
☒	☐	A description of all covariates tested
☒	☐	A description of any assumptions or corrections, such as tests of normality and adjustment for multiple comparisons
☐	☒	A full description of the statistical parameters including central tendency (e.g. means) or other basic estimates (e.g. regression coefficient) AND variation (e.g. standard deviation) or associated estimates of uncertainty (e.g. confidence intervals)
☐	☒	For null hypothesis testing, the test statistic (e.g. F, t, r) with confidence intervals, effect sizes, degrees of freedom and P value noted<br>*Give P values as exact values whenever suitable.*
☒	☐	For Bayesian analysis, information on the choice of priors and Markov chain Monte Carlo settings
☒	☐	For hierarchical and complex designs, identification of the appropriate level for tests and full reporting of outcomes
☒	☐	Estimates of effect sizes (e.g. Cohen's *d*, Pearson's *r*), indicating how they were calculated

Our web collection on statistics for biologists contains articles on many of the points above.

Software and code

Policy information about availability of computer code

Data collection	Illumina HiSeq 3000 PE150 (BSR-seq, RNA-seq, Shallow whole genome sequencing); QuantStudio™ Design & Analysis Software_1.5.2 (Quantitative PCR); ImageJ 1.53f51 (photo cropping); AlphaImager HP_3.4.0; Tecan i-control_3.1.9.0.
Data analysis	Microsoft Excel 2016 (two-side Student's t-test, one-way ANOVA and Tukey's test); GraphPad Prism v6.0.7; bwa/0.7.17; fastqc/0.11.7l; fastq-stats ea-utils/1.1.2; GenomeAnalysisTK/4.0.4.0; picard-tools/2.18.11 & 2.9.0; samtools/1.8; SnpEff/4.3t; STAR/2.6.0c; subread/1.6.0; trimmomatic/0.36; vcftools/0.1.15; R/3.4.2; R/3.3.3; DESeq2_1.36.0; dplyr_0.8.3; ggplot2_3.2.0; optparse_3.2; pheatmap_1.0.12; PoiClaClu_1.0.2.1; RColorBrewer_1.1-2; tidyr_0.8.3; ImageJ 1.53f51 (leaf necrosis analysis); MEGA-X v10.2.4(phylogenetic analysis, with MUSCLE built-in).<br>Code availability<br>All scripts used in this paper have been deposited on Github: https://github.com/Kuhlemeier-lab/Petunia_hybrid_necrosis.

For manuscripts utilizing custom algorithms or software that are central to the research but not yet described in published literature, software must be made available to editors and reviewers. We strongly encourage code deposition in a community repository (e.g. GitHub). See the Nature Portfolio guidelines for submitting code & software for further information.

Data

Policy information about availability of data

All manuscripts must include a data availability statement. This statement should provide the following information, where applicable:

- Accession codes, unique identifiers, or web links for publicly available datasets
- A description of any restrictions on data availability
- For clinical datasets or third party data, please ensure that the statement adheres to our policy

BSR-seq reads have been deposited in the NCBI Sequence Read Archive (SRA) under BioProject PRJNA708139 (https://dataview.ncbi.nlm.nih.gov/object/PRJNA708139). Shallow whole-genome sequencing reads data have been deposited under BioProject PRJNA705072 (https://dataview.ncbi.nlm.nih.gov/object/PRJNA705072). RNA-seq reads have been deposited under BioProject PRJNA705649 (https://dataview.ncbi.nlm.nih.gov/object/PRJNA705649). The Petunia axillaris N 4.03 genome assembly has been deposited at NCBI GenBank under the accession JANRMM000000000 (https://www.ncbi.nlm.nih.gov/bioproject/?term=JANRMM000000000).

Human research participants

Policy information about studies involving human research participants and Sex and Gender in Research.

Reporting on sex and gender	N/A
Population characteristics	N/A
Recruitment	N/A
Ethics oversight	N/A

Note that full information on the approval of the study protocol must also be provided in the manuscript.

Field-specific reporting

Please select the one below that is the best fit for your research. If you are not sure, read the appropriate sections before making your selection.

☒ Life sciences ☐ Behavioural & social sciences ☐ Ecological, evolutionary & environmental sciences

For a reference copy of the document with all sections, see nature.com/documents/nr-reporting-summary-flat.pdf

Life sciences study design

All studies must disclose on these points even when the disclosure is negative.

Sample size

No statistical methods were used to predetermine sample size. Sample size was selected based on previous studies: we followed as (Esfeld et al., Current Biology, 2018) for the sample size of VIGS treatment. We followed as (Chae et al., Cell, 2014) for the sample size of transient overexpression analysis. We followed as (Hemetsberger et al., 2012) for the sample size of DAB staining assay. We followed as (Sheehan et al., 2016) for the sample size of the gene expression analysis by quantitative RT-PCR. We followed as (Liu et al., 2014) for the sample size of the chitinase/lysozyme activity tests. All sample sizes are described for each experiment in figures and figure legends.

References
Laitinen, R.A., Rowan, B.A., Tenenboim, H., Lechner, S., Demar, M., Habring-Muller, A., Lanz, C., Ratsch, G., and Weigel, D. (2014). Species-wide genetic incompatibility analysis identifies immune genes as hot spots of deleterious epistasis. Cell 159, 1341-1351.
Esfeld, K., Berardi, A.E., Moser, M., Bossolini, E., Freitas, L., and Kuhlemeier, C. (2018). Pseudogenization and Resurrection of a Speciation Gene. Curr Biol 28, 3776-3786 e3777.
Hemetsberger, C., Herrberger, C., Zechmann, B., Hillmer, M., and Doehlemann, G. (2012). The Ustilago maydis effector Pep1 suppresses plant immunity by inhibition of host peroxidase activity. PLoS Pathog 8, e1002684.
Liu, X., Grabherr, H.M., Willmann, R., Kolb, D., Brunner, F., Bertsche, U., Kuhner, D., Franz-Wachtel, M., Amin, B., Felix, G., Ongena, M., Nurnberger, T., and Gust, A.A. (2014). Host-induced bacterial cell wall decomposition mediates pattern-triggered immunity in Arabidopsis. Elife 3.
Sheehan, H., Moser, M., Klahre, U., Esfeld, K., Dell'Olivo, A., Mandel, T., Metzger, S., Vandenbussche, M., Freitas, L., and Kuhlemeier, C. (2016). MYB-FL controls gain and loss of floral UV absorbance, a key trait affecting pollinator preference and reproductive isolation. Nat Genet 48, 159-166.

Data exclusions

No data was excluded from the analyses.

Replication

All experiments in this study were repeated independently at least three times with similar results. The number of replications is shown in figure legends.

Randomization	All samples were arranged randomly into experimental groups.
Blinding	Bias could not be introduced since samples were treated identically and collected randomly. Blind was not applied.

Reporting for specific materials, systems and methods

We require information from authors about some types of materials, experimental systems and methods used in many studies. Here, indicate whether each material, system or method listed is relevant to your study. If you are not sure if a list item applies to your research, read the appropriate section before selecting a response.

Materials & experimental systems

n/a	Involved in the study
☒	☐ Antibodies
☒	☐ Eukaryotic cell lines
☒	☐ Palaeontology and archaeology
☒	☐ Animals and other organisms
☒	☐ Clinical data
☒	☐ Dual use research of concern

Methods

n/a	Involved in the study
☒	☐ ChIP-seq
☒	☐ Flow cytometry
☒	☐ MRI-based neuroimaging
